# E-Selectin Ligands in the Human Mononuclear Phagocyte System: Implications for Infection, Inflammation, and Immunotherapy

**DOI:** 10.3389/fimmu.2017.01878

**Published:** 2018-01-19

**Authors:** Mariana Silva, Paula A. Videira, Robert Sackstein

**Affiliations:** ^1^Department of Dermatology, Harvard Skin Disease Research Center, Brigham and Women’s Hospital, Harvard Medical School, Boston, MA, United States; ^2^Program of Excellence in Glycosciences, Harvard Medical School, Boston, MA, United States; ^3^UCIBIO, Departamento Ciências da Vida, Faculdade de Ciências e Tecnologia, Universidade NOVA de Lisboa, Lisboa, Portugal; ^4^Professionals and Patient Associations International Network (CDG & Allies – PPAIN), Faculdade de Ciências e Tecnologia, Universidade NOVA de Lisboa, Lisboa, Portugal; ^5^Department of Medicine, Brigham and Women’s Hospital, Harvard Medical School, Boston, MA, United States

**Keywords:** mononuclear phagocyte, HCELL, E-selectin ligand, cell migration, E-selectin, sialyl Lewis X

## Abstract

The mononuclear phagocyte system comprises a network of circulating monocytes and dendritic cells (DCs), and “histiocytes” (tissue-resident macrophages and DCs) that are derived in part from blood-borne monocytes and DCs. The capacity of circulating monocytes and DCs to function as the body’s first-line defense against offending pathogens greatly depends on their ability to egress the bloodstream and infiltrate inflammatory sites. Extravasation involves a sequence of coordinated molecular events and is initiated by E-selectin-mediated deceleration of the circulating leukocytes onto microvascular endothelial cells of the target tissue. E-selectin is inducibly expressed by cytokines (tumor necrosis factor-α and IL-1β) on inflamed endothelium, and binds to sialofucosylated glycan determinants displayed on protein and lipid scaffolds of blood cells. Efficient extravasation of circulating monocytes and DCs to inflamed tissues is crucial in facilitating an effective immune response, but also fuels the immunopathology of several inflammatory disorders. Thus, insights into the structural and functional properties of the E-selectin ligands expressed by different monocyte and DC populations is key to understanding the biology of protective immunity and the pathobiology of several acute and chronic inflammatory diseases. This review will address the role of E-selectin in recruitment of human circulating monocytes and DCs to sites of tissue injury/inflammation, the structural biology of the E-selectin ligands expressed by these cells, and the molecular effectors that shape E-selectin ligand cell-specific display. In addition, therapeutic approaches targeting E-selectin receptor/ligand interactions, which can be used to boost host defense or, conversely, to dampen pathological inflammatory conditions, will also be discussed.

## Introduction

The mononuclear phagocyte system (MPS) comprises monocytes, dendritic cells (DC), and tissue-resident macrophages. MPS cells have specialized phagocytic capabilities, and antigen processing and presenting functions, thereby initiating the immune response and linking innate and adaptive immune systems (1). In addition to their role as key sentinels and regulators of immunity, mononuclear phagocytes are also involved in several pathological inflammatory conditions, including autoimmune diseases, infection, cancer, and abnormal wound healing processes (2). To access inflammatory sites, circulating monocytes and DCs must first engage the vascular endothelial barrier against the prevailing forces of hemodynamic shear, a process that occurs *via* adhesive interactions between vascular E-selectin and its glycan counter-receptors (E-selectin ligands) on the circulating cells (3). This initial contact results in tethering and slow rolling of the cells along the endothelial surface at velocities well below that of blood flow (4). E-selectin-mediated slow rolling is a vital step in this cascade of events as it allows intimate contact between MPS cells and the inflamed endothelium, and the recognition of inflammatory molecules within the milieu (3). Consequently, a greater knowledge of how E-selectin ligand display is elaborated by different types of circulating monocytes and DCs is key to understanding the physiological and pathological events associated with the MPS. In this review, we will provide information on the structural biology and operation of the wide variety of E-selectin-binding glycoconjugates expressed by circulating MPS cells (i.e., blood monocyte and non-tissue-resident DC populations) in light of their impact on pathology and potential therapies. Furthermore, we will discuss the molecular basis of the biosynthesis of these glycoconjugates, and how such knowledge can frame novel strategies to inhibit or enforce trafficking of MPS cells.

## Mononuclear Phagocyte Family: Heterogeneity and Migratory Capabilities

### Monocytes

**Figure 1:**
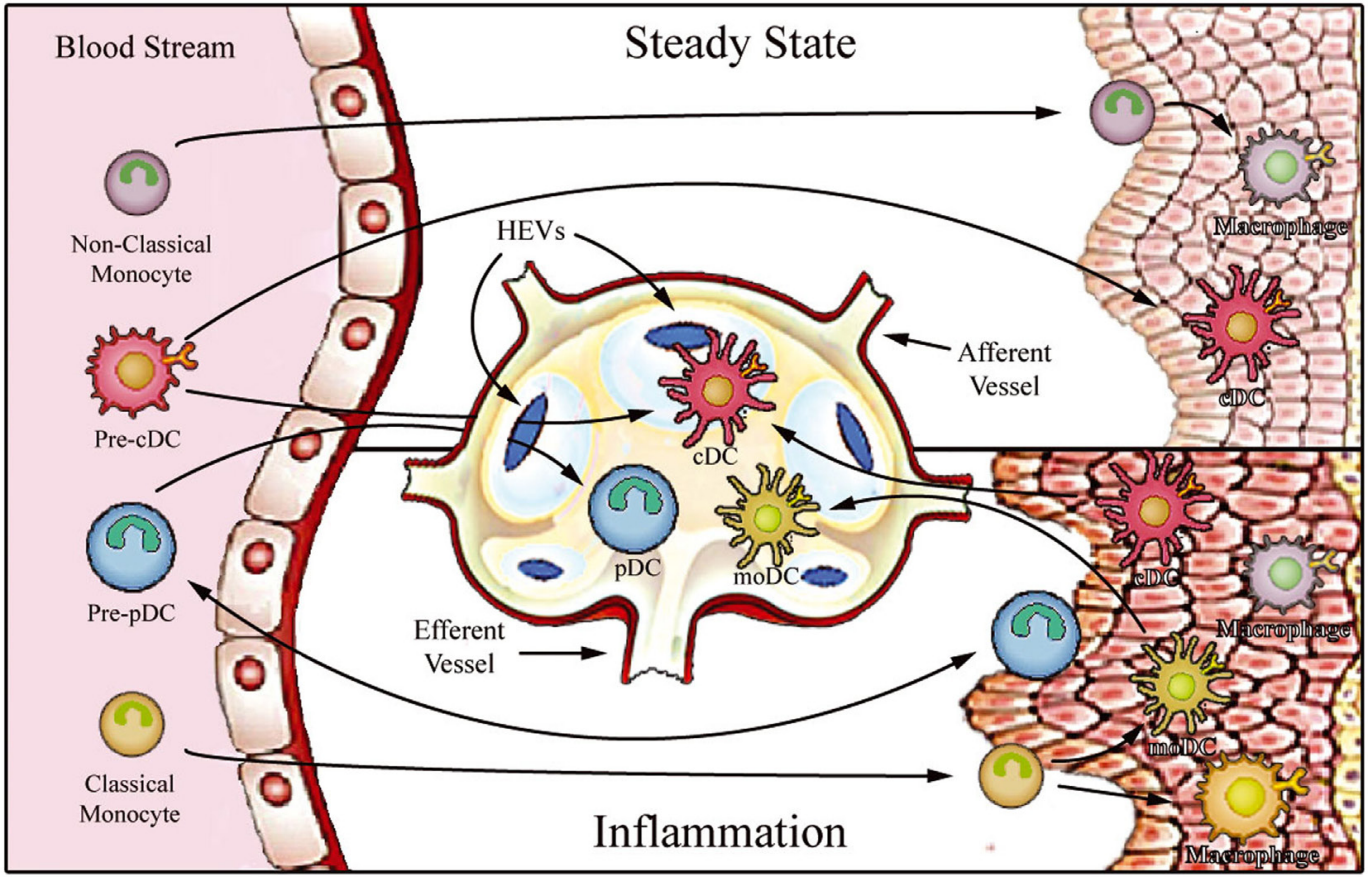
Proposed model for migration of human monocytes and dendritic cell (DC) progenitors into tissues in steady-state and inflammatory conditions. After differentiation in the bone barrow, precursors of DCs and monocytes enter the blood stream and are distributed to lymphoid organs [through high endothelial venules (HEV)] and to various peripheral tissues. In steady state, non-classical monocytes are preferentially recruited into the resting vasculature, where they patrol the endothelium and may contribute to the maintenance of tissue-resident macrophage and DC populations. Conventional DCs (cDCs) recirculate between peripheral tissues and lymphoid organs (migratory cDCs), participating in the induction of peripheral tolerance, or reside in the lymphoid organs (lymphoid-resident cDCs). By contrast, plasmacytoid DCs (pDCs) mostly populate lymphoid tissues (lymphoid-resident pDCs) and lack migratory ability under steady-state conditions. Upon inflammation, classical monocytes, cDCs, and pDCs are recruited to affected tissues. After antigen uptake and differentiation into fully functional mature DCs, monocyte-derived DCs (moDCs), and cDCs enter draining lymph nodes *via* afferent lymphatics. pDCs can only access reactive lymph nodes from the blood stream *via* HEVs.

Monocytes constitute a heterogeneous cell population, comprising approximately 5–10% of total peripheral blood leukocytes. These cells arise from granulocyte–macrophage progenitors in the bone marrow and are subsequently released into peripheral blood, where they circulate for several days (5). At steady state (i.e., without any inflammatory cue), monocytes can enter non-lymphoid tissues, and there they either retain their blood monocytic behavior (6), or generate the immediate precursors of “monocyte-derived macrophages and DCs,” which constitute a small portion of tissue-resident macrophage and DC populations (7–9). On the other hand, under inflammatory conditions, monocytes transmigrate into injured tissues, where they then directly mediate antimicrobial activity or, depending on the local biochemical milieu, differentiate into inflammatory macrophages or monocyte-derived DCs (moDCs) (10) (Figure 1). Circulating monocytes, thus, function as a systemic reservoir of tissue-resident myeloid cells (11, 12).

There are three subsets of human monocytes, each of which display different functional and migratory abilities and can be distinguished based on their expression of specific chemokine receptors, CD14 [the lipopolysaccharide (LPS) receptor], and CD16 (Fcγ RIII) (13). “Classical” monocytes (CD14^++^CD16^−^), which account for about 90% of circulating monocytes in healthy individuals, express high levels of the C-C chemokine receptor type 2 (CCR2), display high phagocytic and myeloperoxidase activities, generate reactive oxygen species, and produce inflammatory cytokines, such as interleukin (IL)-1β, IL-6, and tumor necrosis factor (TNF)-α (14). On the other hand, the “non-classical” monocytes (CD14^+^CD16^++^) comprise a population that exhibits low phagocytic and myeloperoxidase activities (15, 16). Importantly, while classical monocytes are recruited preferentially to distressed tissues (17), non-classical monocytes are recruited to non-inflamed areas, where they patrol the microvasculature *via* the CX3C chemokine receptor 1 (CX3CR1) and leukocyte function-associated antigen (LFA)-1, monitoring the luminal surface of resting endothelium for signs of tissue damage or infection (18–20). In addition, non-classical monocytes are mainly responsive to virus-associated signals, *via* toll-like receptors (TLRs) 7 and 8, whereas classical monocytes respond mostly to bacteria-associated signals (21). An intermediate subset of monocytes, characterized as CD14^++^CD16^+^, is viewed as being a transitional population between classical and non-classical monocyte subsets, displaying significant production of TNF-α and IL-1β, but low peroxidase activity (16, 22). While the migratory ability of the intermediate subset is controversial, they express the chemokine receptor CCR2, a feature supporting their ability to infiltrate sites of inflammation (23). Still, overall, the intermediate monocyte population reportedly displays weaker ability to migrate across resting endothelium compared to the other two monocytic subsets (24).

### Dendritic Cells

Dendritic cells are the antigen-presenting cells par excellence, showing a unique capacity to initiate immune responses. These specialized antigen-presenting cells constitute a unique leukocyte population that display high morphological and functional heterogeneity (25). DCs can be originated from common myeloid or lymphoid precursors and are divided into two main groups: conventional DCs (cDCs) and plasmacytoid DCs (pDCs) (26). After being released into the bloodstream, they are distributed to lymphoid organs (lymph nodes, spleen, and thymus) and various peripheral tissues. DC function is intrinsically related to their anatomical localization, and, therefore, a stringent DC functional-anatomical classification needs to be defined (Figure 1). At steady state, DCs are found to be immature (as indicated by high phagocytic and endocytic capacity and low expression of MHC and costimulatory molecules) and can be classified as either migratory or lymphoid-resident DCs (27, 28). Migratory DCs serve as immune sentinels screening peripheral tissues for signals of danger. They can also capture apoptotic cells or self-antigens in non-inflamed tissues and, after entering lymph nodes *via* afferent lymphatics, present these to T cells in the lymph nodes, thus playing a key role in antigen-mediated peripheral tolerance (29–31). On the other hand, lymphoid-resident DCs differentiate within lymphoid organs directly from blood DC precursors, and they function to continuously survey blood or lymph (27, 32). Both cDC and pDC hematopoietic progenitors contribute to the lymphoid-resident DC pool, whereas most migratory DCs arise from blood cDCs (33). Under infection or sterile inflammatory circumstances, both circulating cDCs and classical monocytes enter inflamed tissues, where they capture antigens and differentiate into highly functional mature DCs. The mature DCs migrate to the lymph nodes *via* the afferent lymph, initiating T cell-mediated immune responses.

In addition to cDCs and pDCs, a distinct subset of DCs are derived from monocytes (known as “moDCs”) which are considered to be “inflammatory DCs”; these cells prominently produce TNF-α, nitric oxide, and IL-23, and are potent inducers of TH17 cells (34–37). Interestingly, although pDCs are believed to be absent from peripheral tissues under steady-state conditions, a number of recent publications reported pDC extravasation into some inflamed tissues, where they secrete large amounts of type I interferon (38–42). In contrast to cDCs, pDCs do not enter reactive secondary lymphoid organs after trafficking from peripheral tissue *via* afferent lymphatics; instead, they apparently migrate directly from the bloodstream *via* high endothelial venules (HEVs) by an E-selectin-dependent mechanism (43–47).

### Macrophages

Macrophages are a heterogeneous and versatile population of tissue-resident cells, mostly originating from self-renewing embryo-derived progenitors and from blood monocytes that have colonized tissues (48, 49). They exist virtually in every tissue throughout the body, where they survey for potential signs of infection/danger and perform phagocytic clearance of dying cells (50). In addition, macrophages play a role in adaptive immunity through antigen presentation and production of cytokines (51, 52).

There are two main macrophage subsets, the M1 and the M2 macrophages, with distinct responses to environmental signals. The M1 subset produces high amounts of pro-inflammatory cytokines and reactive oxygen and nitrogen species, thus playing a crucial role in Th1 polarization and promotion of cellular immunity. M2 macrophages are characterized by their ability to stimulate humoral immune responses, fight extracellular parasite infections, and promote tissue repair, angiogenesis, and tumor progression (53, 54). Whereas the major function of macrophages is to fight infections and kill target cells, they do not typically display hematogenous migration, nor leave sites of tissue injury (11, 55).

### MPS Extravasation Cascade: The Multistep Model

Recruitment of circulating cells from blood to inflamed tissue involves a sequential and coordinated series of molecular actions mediated by adhesive interactions between circulating sentinels and endothelial cells in post-capillary venules (56). Here, we review the molecular effectors that regulate the initial phagocyte–endothelial binding interactions, which are essential for transendothelial migration of blood monocytes and DCs to sites of injury.

**Figure 2:**
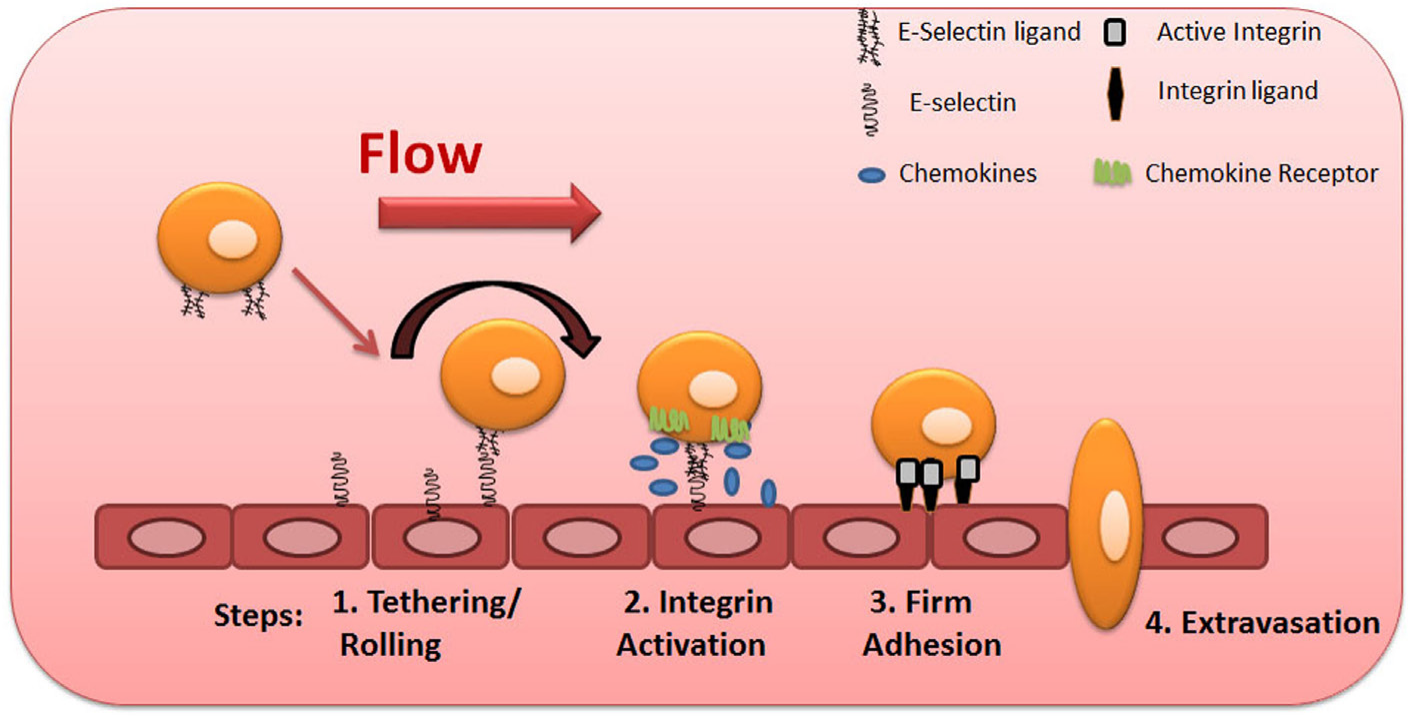
Multistep model of circulating blood cell adhesion and migration along the vascular endothelium. Cells make adhesive contacts onto the inflamed endothelial surface through engagement of their sialofucosylated glycan determinants to vascular E-selectin (Step 1—tethering and rolling). Subsequent engagement of chemokine receptors leads to integrin activation (Step 2) and firm adhesion of leukocytes to endothelium (Step 3), allowing their transmigration (Step 4).

To initiate the extravasation process, circulating phagocytes establish low-affinity and reversible interactions (tethering) on target endothelial cells, achieving low velocity “rolling” adhesive interactions (Step 1, Figure 2). Rolling exposes these cells to chemokines that are immobilized by glycosaminoglycans on the endothelial surface, and, in turn, facilitates engagement of G-protein-coupled chemokine receptors (GPCRs) expressed on the mononuclear phagocyte cell surface (Step 2, Figure 2), with resultant G-protein-driven integrin activation (3, 57). Activated integrins on phagocytes, principally very late activation protein 4 and LFA-1, bind to their respective endothelial receptors vascular cell adhesion molecule-1 and intercellular adhesion molecule-1 (ICAM-1), leading to firm adhesion of MPS cells on the endothelium (Step 3, Figure 2) (3, 57). The binding of activated integrins then allows diapedesis into the tissue (Step 4, Figure 2). Two distinct mechanisms enable diapedesis: (1) transient dismantling of endothelial junctions (paracellular migration) or (2) migration through individual endothelial cells (transcellular migration) (58, 59).

Although several cell-associated proteins are specialized at mediating the first step of cell migration, the selectins and their ligands are the most potent effectors of tethering and rolling adhesive interactions. These molecules are responsible for the initial low-affinity binding interactions of leukocytes on endothelial layer (60), a property related to the unique biophysics of lectin–carbohydrate interactions under fluid shear conditions.

## Selectins and Their Glycoconjugate Ligands

### The Selectin Family

**Figure 3:**
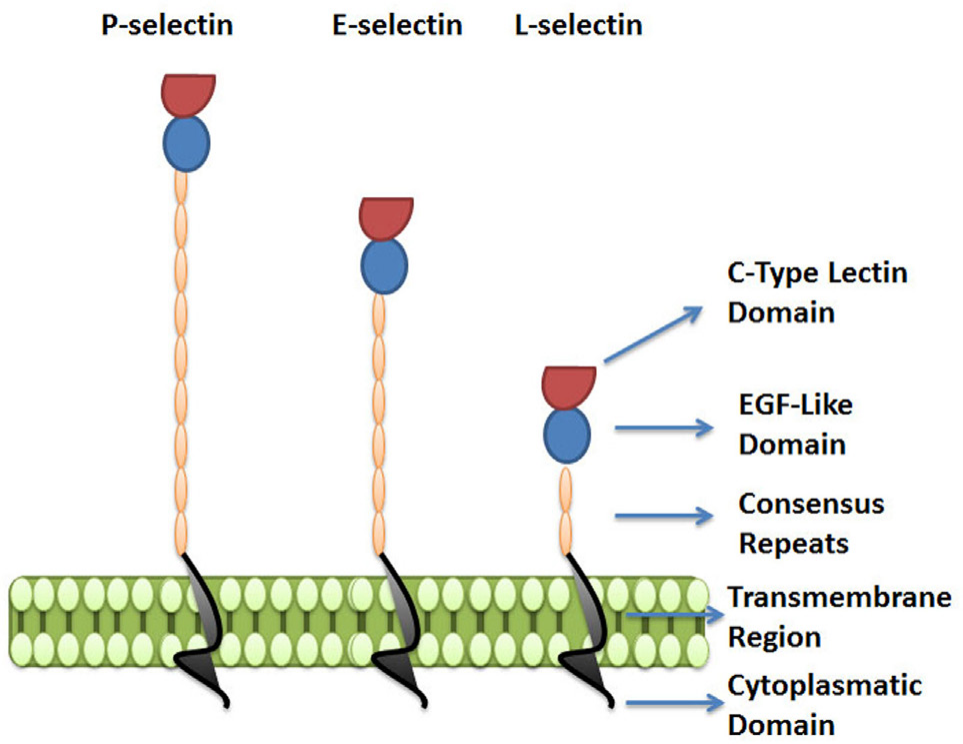
The selectin family. Selectins are a family of three carbohydrate-binding proteins: P-selectin, expressed on activated platelets and endothelial cells, E-selectin expressed on activated endothelial cells, and L-selectin expressed on leukocytes. The figure represents the five domains shared by selectins: C-type lectin domain, epidermal growth factor-like domain (EGF), a varying number of short consensus repeats having homology to complement regulatory proteins, a transmembrane region, and a cytoplasmatic domain.

The selectins are a family of three carbohydrate-binding proteins that can be expressed on endothelial cells, leukocytes and platelets (Figure 3). Due to their requirement of calcium ions for binding, all three selectins, E-selectin (CD62E), P-selectin (CD62P), and L-selectin (CD62L), belong to the C-type lectin family (61). Selectins share a common structure of five different domains: an *N*-terminal carbohydrate recognition domain (CRD), an epidermal growth factor-like domain (EGF), a varying number of short consensus repeats that have homology to complement regulatory domains (“CRs” of which there are 2, 6, and 9 within L-, E-, and P-selectin, respectively), a transmembrane region, and a C-terminal cytoplasmatic domain (Figure 3) (62–64). While the CRD and EGF domains are highly homologous between the three selectins, the structure of the transmembrane and cytoplasmic portions, as well as the extracellular CR domains are not conserved across the selectins, resulting in structural diversity and varying molecular weights between selectins (61, 65).

Despite sharing common elements, the three selectins have different functions in diverse pathological and physiological processes and vary in their distribution and binding kinetics.

The biology of L-selectin was first elucidated by use of an *in vitro* assay in which suspensions of lymphocytes were overlaid onto lymph node sections (66). This assay then allowed for creation of mAb that could interrupt this binding, such as the mAb known as “MEL-14” described by Gallatin and coworkers (67) in 1983, and thereafter led investigators to cloning of this structure (62). L-selectin is highly expressed on hematopoietic stem cells and mature leukocytes, including all myeloid cells, subsets of natural killer cells, *naïve* T and B cells, and central memory T cells. When leukocytes are activated, cell surface levels of L-selectin are downregulated by proteolytic cleavage *via* metalloprotease-dependent shedding of the extracellular domain (61, 68, 69).

P-selectin was described in 1984 by McEver and coworkers (70, 71) and Furie and coworkers (72) as a glycoprotein expressed on the cell surface of activated platelets. P-selectin is constitutively expressed by circulating platelets and endothelial cells, where it is stored in α-granules and Weibel–Palade bodies, respectively. Because it can be expressed on endothelial cells, P-selectin together with E-selectin (described below) are known as the “vascular selectins.” Following pro-inflammatory stimulus by molecules such as thrombin or histamine, P-selectin is rapidly translocated from the granules to the cell surface by fusion of intracellular storage compartments with the plasma membrane. In murine endothelial cells, inflammatory mediators, such as TNF-α, IL-1β, and LPS, induce P-selectin mRNA transcription, which requires the cooperative binding of the nuclear factor κ-light chain-enhancer of activated B cells (NF-κB) and activating transcription factor-2 (ATF-2) to their response elements within the P-selectin promoter (73–75). However, importantly, the promoter of P-selectin in humans and other primates lacks binding sites for NF-κB and ATF-2 (76). For this reason, in human endothelial cells, the only vascular selectin inducibly expressed by TNF-α, LPS, and IL-1β is E-selectin (77).

E-selectin was first reported by Bevilacqua and coworkers (63, 78) in 1980s as a leukocyte adhesion molecule on activated endothelial cells. Skin and bone marrow microvessels express E-selectin constitutively (79), however, in other tissues, endothelial cells do not constitutively express E-selectin but its expression is strongly upregulated by inflammatory cytokines, such as TNF-α and IL-1β. These cytokines potently induce transient transcription (within hours of exposure) of E-selectin mRNA in both human and mouse endothelial cells (80). Cytokine-dependent activation of E-selectin is mediated by NF-κB binding to regulatory domains in the E-selectin promoter (81). Functionally, E-selectin slows leukocyte rolling to much lower velocities than do either L- or P-selectin, favoring subsequent leukocyte arrest (4, 82). This capacity, along with the inability of human endothelial cells to upregulate P-selectin in the presence of IL-1β and TNF-α, is why E-selectin is considered to be the most important selectin for cell trafficking to sites of inflammation in humans, and it plays a critical role in the recruitment of immune effectors to target inflammatory sites.

### The Carbohydrate E-Selectin Ligands

**Figure 4:**
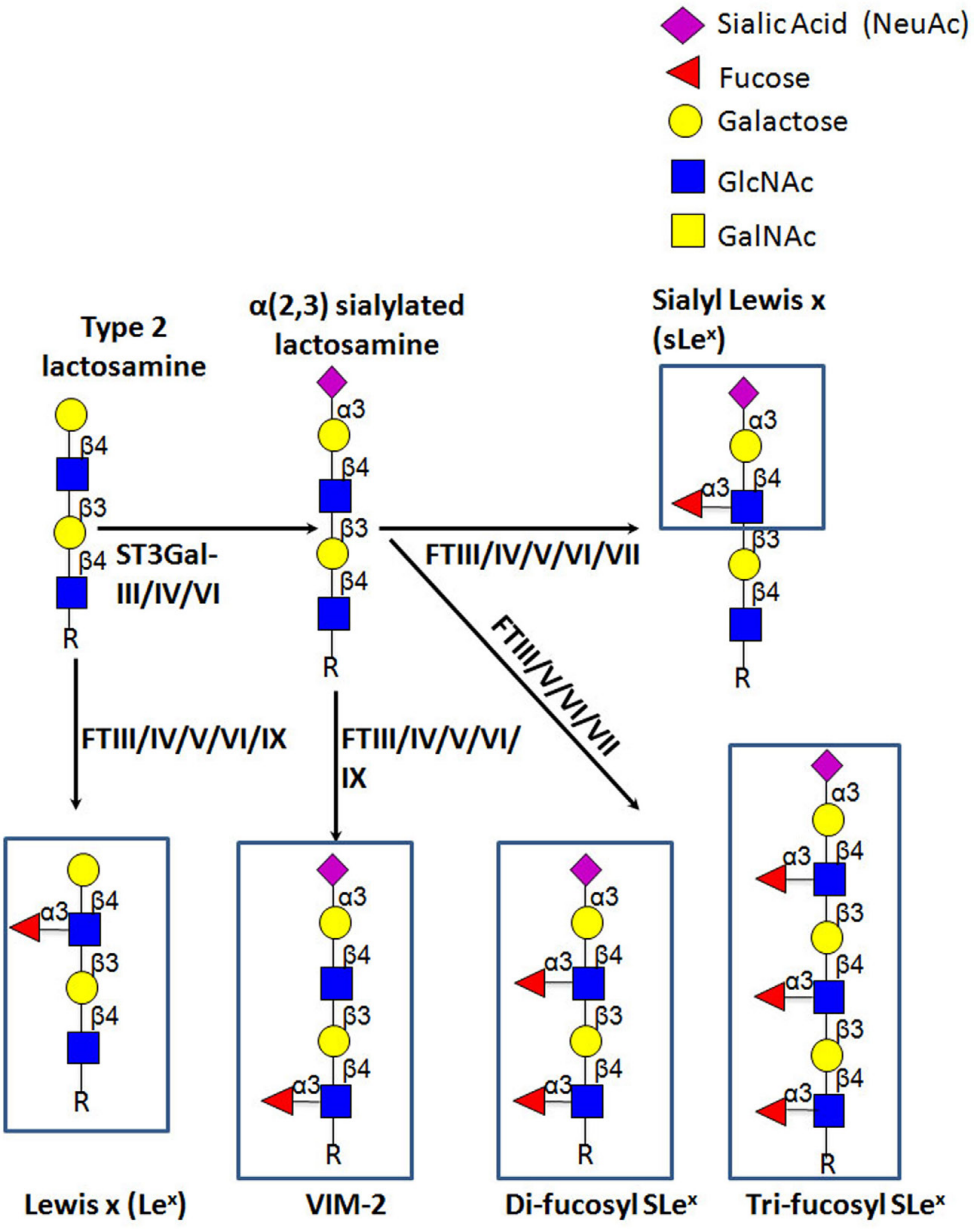
Schematic representation of biosynthesis of the E-selectin ligand determinants. The α(2,3)-sialyltransferases, ST3Gal-III, -IV, and -VI, terminate the elongation of both *O*- and *N*-glycans by creating sialylated type 2 lactosamine chains. These can be further fucosylated by the action of the specific fucosyltransferases, yielding different Lewis-related structures that display E-selectin binding activity.

E-selectin recognizes a range of structurally diverse glycan epitopes expressed by human leukocytes that typically contain α(1,3)-fucose (Fuc) and α(2,3)-sialic acid (Sia) modification(s) on a lactosamine backbone [consisting of galactose (Gal) linked to *N*-acetylglucosamine (GlcNAc)], as shown in Figure 4 (79). The terminal tetrasaccharide known as sialyl Lewis X (sLe^x^—Siaα2-3Galβ1-4(Fucα1-3)GlcNAc) is the prototypical E-selectin-binding determinant (83–85). Some sLe^x^-variant structures can also exhibit E-selectin binding activity, namely an internally fucosylated sLe^x^-variant (VIM-2) and other polylactosamine structures, in which Fuc modifications occur at more than one GlcNAc residue along the polylactosamine chain (tri-fucosyl-sialyl Lewis^x^ and di-fucosyl-sialyl Lewis^x^) (86–88). In addition, other glycan structures that are not natively expressed on leukocytes exhibit E-selectin binding activity, namely the sLe^x^ isomer, sialyl Lewis a (sLe^a^—Siaα2-3Galβ1-3(Fucα1-4)GlcNAc) (89), some sulfated derivatives of Le^x^ and Le^a^ (3′-sulfo-Le^x^ and 3′-sulfo-Le^a^, respectively) (90, 91), and a fucosylated glycoform of LacdiNac that displays a terminal *N*-acetylgalactosamine (GalNAc) instead of Sia (GalNAc-Lewis x) (92).

### Glycosyltransferases Involved in the Biosynthesis of Selectin–Carbohydrate-Binding Determinants

E-selectin binding determinants are typically displayed at the end of *O*-glycans, *N*-glycans, or glycolipid precursor structures, and require the coordinated and sequential action of specific glycosyltransferases localized within the lumen of the Golgi apparatus. Assembly of sLe^x^ is driven by the terminal addition of Sia (to Gal) and of Fuc (to GlcNAc) through the action of α(2,3)-sialyltransferases and α(1,3)-fucosyltransferases (FTs), respectively, on type 2 lactosamine (LacNAc) chains (i.e., Gal connected to GlcNAc through a β(1,4)-linkage) (Figure 4) (93, 94). The sialylated forms of Lewis antigens are synthesized by the action of the α(2,3)-sialyltransferases (ST3Gal isoenzymes). These enzymes transfer Sia residues to the Gal on the LacNAc chain, exclusively acting prior to fucosylation (95, 96). There are six members of the α(2,3)-sialyltransferase family (ST3Gal-I–ST3Gal-VI), but only ST3Gal-III, ST3Gal-IV, and ST3Gal-VI are reported to sialylate lactosamine chains (97). Importantly, ST3Gal-III exhibits preference for type 1 lactosamine chain acceptors (wherein Gal is connected to GlcNAc through a β(1,3)-linkage), whereas ST3Gal-IV and ST3Gal-VI preferentially act on type 2 polylactosamine chains (98–100). When Type 1 lactosamines are decorated with Sia in α(2,3)-linkage to Gal and with Fuc in β(1,3)-linkage to GlcNAc, this tetrasaccharide is known as sialyl Lewis A (sLe^A^).

So far, six human FTs have been found to catalyze the addition of Fuc at α(1,3) linkage to GlcNAc with a type 2 lactosamine—FTIII, FTIV, FTV, FTVI, FTVII, and FTIX. Each enzyme exhibits specificity for acceptor substrates and, therefore, has the ability to generate distinct fucosylated structures (101, 102). Particularly, FTIII and FTV are unique in that they exhibit both α(1,3) and α(1,4) FT activity on both sialylated and unsialylated type 2 and type 1 lactosamines thereby creating (s)Le^x^ and (s)Le^a^ epitopes, respectively (103, 104). On the other hand, FTIV and FTVI fucosylate both sialylated and unsialylated type 2 lactosamine chains, with FTIV creating VIM-2 and Le^x^ (105, 106) and, modestly, sLe^x^ determinants (107, 108), and FTVI creating these structures as well as di-fucosyl-sLe^x^ (108–110). Uniquely, FTVII can only act on sialylated type 2 lactosamines, yielding sLe^x^ and di/tri-fucosyl-sLe^x^-structures (111, 112), whereas FTIX is known to synthesize mostly Le^x^ (101, 106).

Most of the reports that assess the role of the different glycosyltransferases involved in selectin ligand biosynthesis in leukocytes have been performed using knock-out mouse models, with a small proportion of these studies using human leukocytes or human hematopoietic cell lines. Concerning the role of the α(2,3)-sialyltransferases, murine studies suggest that ST3Gal-IV and ST3Gal-VI collaborate together in murine E-selectin ligand biosynthesis, with ST3Gal-IV having an important role in the regulation of E-selectin-dependent rolling velocity (113, 114). Interestingly, ST3Gal-III does not seem to contribute to the synthesis of murine E-selectin ligand moieties, since deficiency of this enzyme did not affect E-selectin ligand expression or activity on murine leukocytes (113). Surprisingly, ST3Gal-IV is reportedly the only human α(2,3)-sialyltransferase involved in the biosynthesis of E-selectin ligands in human myeloid leukocytes, since ST3Gal-IV-silenced HL-60 cells (a human promyelocytic cell line), and neutrophils derived from stable ST3Gal-IV knockdown hematopoietic stem cells fail to engage in tethering and rolling interactions on E-selectin-bearing substrates (115). In case of α(1,3)-FTs, studies demonstrate that mostly FTVII, and to a lesser extent FTIV, are the key murine α(1,3) FTs that mediate leukocyte selectin ligand biosynthesis. In fact, murine leukocytes lacking FTVII show poor adhesive contacts with E- and P-selectin, indicating that this FT plays a prominent role in murine E-selectin ligand biosynthesis (116, 117). However, others reported that FTIV is crucial for slow murine leukocyte rolling velocity (118, 119). Importantly, E-selectin binding activity conferred by murine FTIV, but not by FTVII, apparently occurs mainly on glycolipids rather than glycoproteins (120). Conversely, in human leukocytes, there is evidence that FTVII, FTIV, and FTIX could each act in synthesis of E-selectin ligand determinants (121). Notably, the mouse genome encodes only FTIV, FTVII, and FTIX (122), whereas primates possess an additional three FT gene products—FTIII, FTV, and FTIV. These additional FTs provide for a much wider capacity to create sLe^X^; in addition, the expression of FTIII and FTV in primates uniquely drives creation of sLe^A^ determinants. Human circulating monocytes express all the α(1,3)-FTs, with the exception of FTV, heightening the potential for variability in glycoconjugates bearing sLe^x^ among human and mouse cells (123). Notably, sLe^A^ is not expressed on any primate leukocytes as these cells do not synthesize Type 1 lactosamines (3).

**Figure 5:**
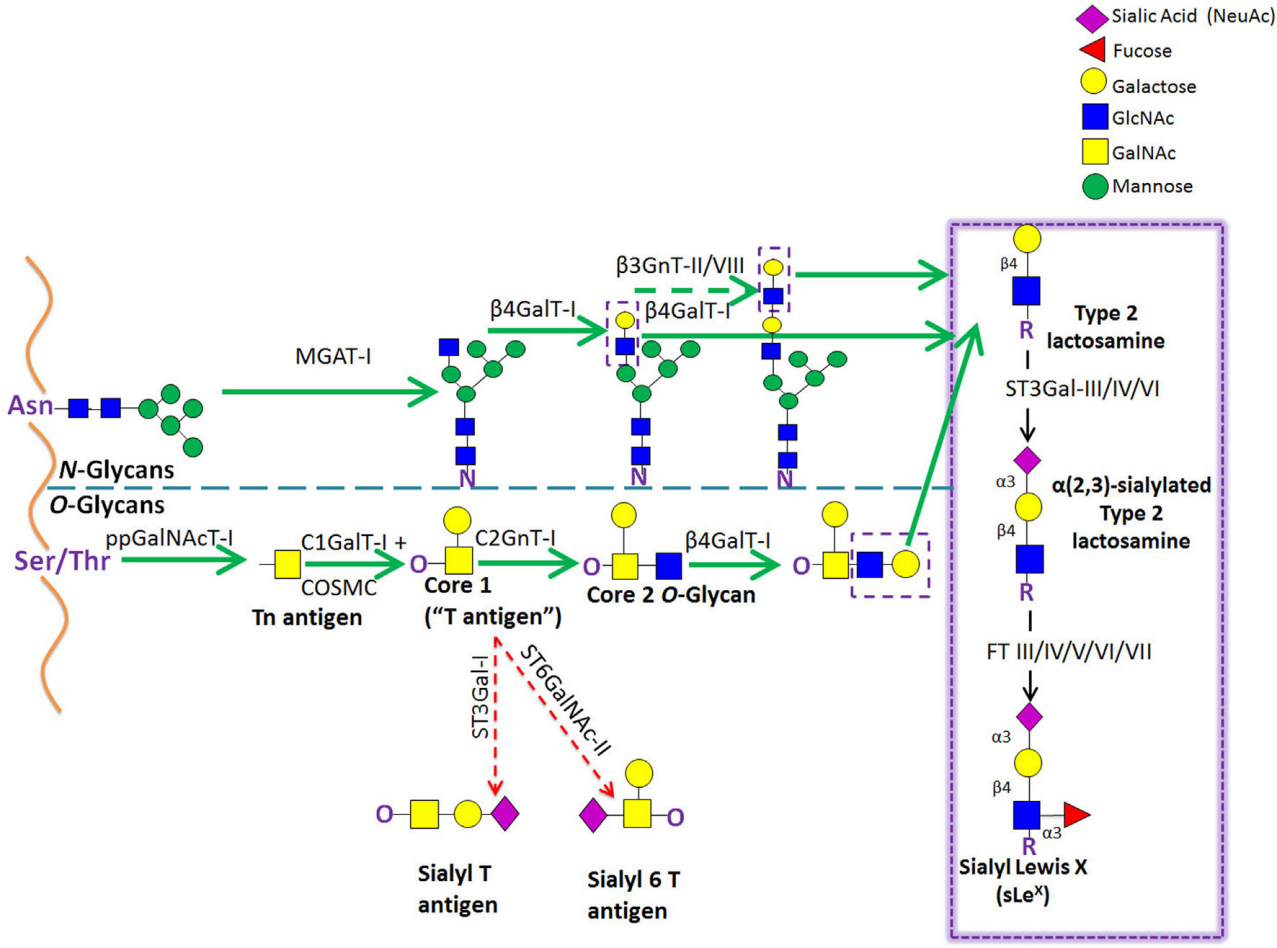
Schematic representation of the biosynthetic pathways leading to glycoprotein synthesis: O-linked and N-linked glycosylation. The *O*-glycosylation process is characterized by a stepwise sugar addition that occurs in the Golgi apparatus and involves a broad array of enzymes. This synthesis is initiated by one of the *N*-acetylgalactosaminyltransferase (ppGalNAcTs) family members, forming the Tn antigen. After the first sugar [*N*-acetylgalactosamine (GalNAc)] addition, Tn is typically elongated by Core 1 β(1,3)galactosyltransferase (Core1GalT or T synthase, whose Golgi expression requires the activity of its chaperone COSMC), creating the “Core 1” *O*-glycan (also known as “T antigen”). Core 1 is then further lengthened by C2GnT-I, which adds an *N*-acetylglucosamine (GlcNAc) to the GalNAc, forming the “Core 2” *O*-glycan structure. Alternatively, Core 1 sialylation, by ST3Gal-I or ST6GalNAc-II [forming sialyl-T (sT) or sialyl-6T (s6T) antigens, respectively] stops Core 2 formation. In contrast to *O*-glycosylation, the *N*-glycosylation process requires the production of an oligosaccharide precursor (GlcNAc2Man5) in the cytoplasmic face of the endoplasmic reticulum (ER) membrane. This glycan flipped in the ER lumen and is then transferred en block from a lipid donor to the Asn residue of a newly synthesized protein within the ER lumen, and then further processed in the Golgi compartment. Biosynthesis of hybrid and complex glycans is initiated by the action of MGAT-I, which adds a GlcNAc residue to the mannose (Man) present on the α(1,3)-arm of the Man5GlcNAc2 structure. Repetitive additions of galactose (Gal) and GlcNAc by β(1/4)GalT and β(1,3)GnT enzymes, respectively, can further elongate Core 2 *O*-glycan and hybrid- and complex-type *N*-glycan structures, creating the lactosaminyl type 2 chains that serve as acceptors for terminal sialofucosylation reactions.

Other glycosyltransferases involved in the biosynthesis of sLe^x^ have also been studied for their relevance in generating functional selectin ligands (Figure 5). Regarding sLe^x^ presentation on O-glycans, one study reported that leukocytes from mice deficient in the enzyme required for initiation of *O*-glycosylation, ppGalNAcT-1, showed impaired recruitment during inflammation due to a significant reduction in E- and P-selectin ligand levels (124). Mice lacking the *O*-glycan core 1 β3galactosyltransferase (C1GalT-I) showed dramatic loss of leukocyte rolling on E-selectin and, consequently, these leukocytes did not transmigrate into inflamed tissues (125). Transgenic mouse studies, where the O-glycan core 2 β6-*N*-acetylglucosaminyltransferase-I (C2GnT-I) was knocked out, also showed reduced E-selectin and P-selectin binding activity of leukocytes under static and shear-based rolling assays, with impaired leukocyte recruitment to sites of inflammation (126–128). In agreement, in human moDCs, the downregulation of C2GnT-I, with concurrent upregulation of ST3Gal-I and GalNAc α(2,6)sialyltransferase (ST6GalNAc)-II, results in a loss of the core 2 structures required for O-glycan display of sLe^x^ (Figure 5) (129). Furthermore, studies using HL-60 cells have revealed that the ST6GalNAc-II overexpression abrogates sLe^x^ cell surface display and reduces the number of adherent cells to E-selectin under flow conditions, reinforcing the notion that there exists a competition between ST6GalNAc-II and C2GnT-I for core 1 acceptors, affecting the biosynthesis of sLe^x^-bearing core 2-*O*-glycan structures (Figure 5) (130). Interestingly, in mice, knockout of one of the β(1,4)galactosyltransferases (of the family of five isoenzymes) involved in Type 2 lactosamine synthesis, β(1,4)galactosyltransferase-I (β(1,4)GalT-I), showed reduced inflammatory responses and impaired P-selectin binding activity; however, the contribution of this enzyme in the synthesis of E-selectin counter-receptors remains to be elucidated (131).

One study has recently evaluated the contributions of *N*-glycans, *O*-glycans and glycosphingolipids (GSLs) to E-selectin binding by human myeloid cells under physiological flow conditions (132). To address this issue, *O*-glycan and GSL synthesis was abolished by, respectively, knocking-out the core 1 Gal transferase chaperone, i.e., the C1GalT-I-specific Molecular Chaperone (COSMC), β1,2 GlcNAc-transferase (MGAT-I), and UDP-glucose ceramide glucosyltransferase (UGCG). Notably, these studies indicate that while *O*-glycans are indispensable for myeloid cell binding to L- and P-selectins, *N*-glycans play the major role in the initial myeloid cell recruitment into E-selectin-bearing substrates, with *O*-glycans playing a more modest role. In addition, both glycolipids and *N*-glycans are responsible for the slowing down of rolling velocities that precede firm arrest (132).

Most studies that have assessed biologic modulators of E-selectin ligands in leukocytes have been performed using human and murine T cells. An array of cytokines has been shown to regulate E-selectin ligand expression *via* upregulation or downregulation of specific glycosyltrasferases that control sLe^x^ expression. Specifically, IL-2, IL-7, IL-15, and IL-12 increase the expression of glycosyltransferases involved in the biosynthesis of E-selectin ligand determinants, whereas IL-4 has the opposite effect (133). This selectin ligand upregulation in T cells in response to cytokine signaling was shown to be dependent on Th1 transcription factor T-bet (134) and on STAT4-mediated pathways (135). Interestingly, human myeloid cells treated with granulocyte-colony stimulating factor (G-CSF) show increased cell surface expression of E-selectin ligands associated with significant increases in gene expression of the glycosyltransferases ST3Gal-IV, FTIV, and FTVII (136).

## E-Selectin Ligand Activity Displayed by Circulating MPS Subsets

Among the cells of MPS, human circulating monocytes and, to a lesser extent, human blood cDCs and moDCs are the most comprehensively analyzed group in terms of E-selectin ligand activity. In our studies, human classical monocytes (CD14^++^CD16^−^) showed significantly higher levels of sLe^x^ determinants as compared to intermediate monocytes (CD14^++^CD16^+^), whereas non-classical monocytes (CD14^+^CD16^++^) were almost devoid of sLe^x^ expression (123). Another study compared the trafficking capacity of human monocyte subsets by analyzing their ability to bind to activated endothelial monolayers, and commensurately, classical monocytes showed noticeably higher capability of adhering to reactive endothelium than did non-classical/intermediate monocytes (137). In agreement with human studies, murine classical monocytes (Ly-6C^hi^) exhibit greater binding to E-selectin under flow conditions and express higher levels of the scaffolds that bear sLe^x^ determinants compared to non-classical monocytes (Ly-6C^lo^) (138, 139). This differential pattern of E-selectin ligand display is in agreement with the specific migratory requirements among the monocyte subsets: classical monocytes are typically recruited to inflamed lesions (138, 139), whereas non-classical monocytes migrate to non-inflamed endothelium (14) upon which they patrol healthy tissues in a LFA-1-dependent manner (19). Indeed, although the first observations of non-classical monocytes were made in non-inflamed skin blood vessels (19), these cells were further described in the microvasculature of kidney under steady-state conditions (20). The patrolling profile that these cells exhibit is independent of the activation state of the endothelium, since non-classical monocytes constitutively scavenge the luminal side of non-reactive endothelium (18). Therefore, their ability to bind to endothelium seems to be independent of E-selectin receptor/ligand interactions, but, instead, appears critically regulated by LFA-1 expression and its interaction with endothelial ICAM (19, 20). Notably, although selectins play a major role in the initial adhesive contacts with endothelium surfaces, integrins can also support tethering and rolling events under flow conditions, albeit with less potency than do selectins (140, 141).

Multiple adhesion molecules are involved in monocyte attachment to endothelium. While E-selectin receptor/ligand interactions prominently mediate Step 1 events in transmigration for all leukocytes, L-selectin-dependent binding interaction have also been observed to potently mediate human peripheral blood monocyte binding to activated vascular endothelium under shear stress (142–144). Thus, even though the majority of the reports indicates that initial monocyte adhesion to activated endothelial cells is most critically dependent on E-selectin receptor/ligand interactions (123, 145–150), distinct interactions were also reported by other authors. The differences have to do with differences in the leukocyte populations under study, variations in the assay conditions employed (i.e., shear stress levels employed, rotatory shear versus fluid shear conditions, temperature, etc.), differences in the adhesion metrics (i.e., number of adhered cells, number of rolling cells, rolling velocity measurements, etc) altogether compounded by the innate biologic differences between mice and human cells, could alternatively emphasize the contribution(s) of other adhesion molecules.

Concerning DCs, human blood cDCs express high levels of sLe^x^ determinants, which allow them to tether and roll on E-selectin under flow conditions (151, 152). Importantly, in *in vivo* intravital microscopy studies, human blood cDCs adoptively transferred into mice were observed to roll along resting murine skin endothelium and extravasate at sites of inflammation (151). Notably, human moDCs significantly express sLe^x^, especially on *O*-glycan structures. Upon maturation of moDCs with the TLR4 ligand, LPS, sLe^x^ expression is downregulated due to decreased C2GnT-I expression and upregulation of ST6GalNAc-II and ST3Gal-I (129). Biologically, these observations suggest that sLe^x^ is less relevant for transendothelial migration of TLR4-induced-mature moDCs, or that maturation is a step that follows transendothelial migration. By contrast, IFN-γ-induced maturation of moDCs leads to an upregulation of C2GnT-I, resulting in increased expression of core 2 *O*-glycan substrates for sLe^x^ decoration (153). These features suggest that sLe^x^-bearing core 1-derived (or core 2) *O*-glycans are required for human moDC migration and are modulated according to specific maturation stimuli. Human pDCs also express sLe^x^, allowing their recruitment to some inflamed tissues (38, 39, 154) and to reactive lymph nodes (43), a process believed to be mediated by the expression of E-selectin in HEVs (45, 47).

## Glycoconjugate Structures That Display E-Selectin Ligand Determinants in Circulating MPS Cells

Several diverse and structurally singular glycostructures with E-selectin binding activity have been identified on human classical monocytes or human blood DCs. Human classical monocytes greatly display sLe^x^ decorations on an array of protein scaffolds, consisting of P-selectin glycoprotein ligand-1 (PSGL-1), CD43 and CD44, and GSLs (123), whereas human circulating DCs appear to display sLe^x^ solely on PSGL-1 (129, 155).

### Cutaneous Lymphocyte Antigen

The cutaneous lymphocyte antigen (CLA) is the E-selectin-reactive glycoform of PSGL-1. PSGL-1 is a transmembrane 240-kDa homodimeric, mucin-like glycoprotein expressed on leukocytes (and, reportedly, on some activated endothelial cells) that plays a crucial role in the homing of leukocytes into inflamed tissue (156, 157). E-selectin binding activity of PSGL-1 is conferred by sialylated and fucosylated core 2-based-*O*-glycans that are cluster-distributed along the stalk region of the PSGL-1 extracellular domain (158). A number of studies have identified PSGL-1 as one of the several scaffolds expressed by human classical monocytes displaying E-selectin-binding activity (123, 155). On the other hand, PSGL-1 is the only known scaffold that presents sLe^x^ determinants on human circulating cDCs (151, 155) and moDCs (129). Yet, Silva et al. observed that although PSGL-1 is essential for P- and L-selectin recognition by human moDCs under fluid shear conditions, it is not mandatory for tethering to E-selectin (153). Moreover, there are reports that extravasation of murine immature DC to inflamed tissues requires both E- and P-selectin, but not PSGL-1 (159). Together, these data suggest the expression of ligands for E-selectin in addition to CLA by human and murine DCs. Still, PSGL-1 is the dominant ligand for P- and L- selectin and is the only known glycoprotein that binds all three selectins (160, 161). Accordingly, circulating monocytes that have already bound to E-selectin on inflamed endothelium can also interact with L-selectin expressed by other circulating monocytes/DCs *via* PSGL-1 and support their secondary capture, potentiating mononuclear phagocyte recruitment to sites of inflammation (162).

### Hematopoietic Cell E- and L-Selectin Ligand

HCELL is a sialofucosylated glycoform of CD44 that exhibits potent E-selectin (and L-selectin) binding activity. CD44 is a transmembrane protein that exists in a wide variety of protein isoforms due to alternative splicing and extensive post-translational modifications (with molecular weight ranging from 80 to 220 kDa). CD44 is expressed by most mammalian cells, where it serves as the principal receptor for hyaloronic acid and participates in a broad range of cellular activities, including lymphocyte activation, leukocyte trafficking, hematopoiesis, cell growth and survival, and tumor dissemination (163). Post-translational modifications along with extensive alternative splicing allow the formation of multiple protein isoforms, expressed in a tissue-specific manner (164, 165). The standard protein isoform of CD44 (CD44s or CD44H) is encoded by mRNA transcripts comprising exons 1–5 and 16–20 (“s1–s5 and s6–s10”). CD44s is ubiquitously expressed by mammalian cells and is the form most often displayed by hematopoietic-lineage cells. In addition to CD44s, non-hematopoietic cells characteristically display CD44 variant isoforms contain peptide products of variant exons (exons “v2–v10”) in addition to the standard exon peptide products.

CD44 post-translational modifications include the addition of different glycan structures, namely glycosaminoglycans, and *N*- and *O*-glycan substitutions (166). While previous studies indicated that HCELL was only expressed by human hematopoietic stem and progenitor cells (HSPCs) (167, 168), and some hematologic (167, 169) and solid malignancies (170), HCELL was recently reported to be expressed by classical human monocytes (123). Importantly, for human HSPCs, the sLe^x^ determinant is exclusively displayed on *N*-glycan lactosamines on CD44s, but classical monocytes express sLe^x^ on *O*-glycans of CD44s (123). Because of its ability to engage E- and L-selectin under relatively high fluid shear conditions (i.e., in excess of 20 dynes/cm^2^ shear stress), HCELL is considered the most potent L- and E-selectin ligand expressed on mammalian cells (167, 171).

### CD43E

CD43, also known as sialophorin or leukosialin, is a cell surface glycoprotein expressed by nearly all hematopoietic cells and is involved in several important processes, including cell development, activation, survival, and migration (172–176). Glycosylation of CD43 molecules with either core 1 or core 2 *O*-glycan structures produces the ~115 and ~135 kDa glycoforms, respectively (177). Recently, we reported that human classical monocytes display E-selectin binding activity on CD43 (123). CD43 that displays sLe^x^ and binds E-selectin is known as “CD43E” and this protein harbors sLe^x^ on *O*-glycans (178).

### Glycosphingolipids

In contrast to results in mouse leukocytes, reports using human cells have suggested that sialofucosylated determinants displayed on lipid scaffolds can mediate adhesion to E-selectin under static and flow conditions. In native human leukocytes and in the myelocytic leukemia cell line HL-60, the glycolipid structures that display ability to bind to E-selectin consist of sialylated lactosylceramides decorated with internal fucosylated GlcNAc structures (myeloglycans) (86, 87, 179). It has been reported that the disruption of myeloglycan biosynthesis *via* knockdown of ceramide glucosyltransferase (UGCG) in HL-60 cells disturbed stable cell rolling and impaired transmigration across inflamed endothelial cells (180). Finally, human classical monocytes treated with a broadly active protease did not show a complete abrogation of sLe^x^ staining, suggesting that although the majority of sialofucosylated moieties required for E-selectin binding are preferentially expressed on proteins rather than lipids, a considerable amount of sialofucosylated determinants are present on glycolipids (123).

## Pathophysiological Importance of the Selectins

Due to its crucial role in the transmigration of specific myeloid populations to sites of inflammation, the selectin/selectin–ligand axis is involved in the development of many acute and chronic inflammatory conditions (181, 182). In fact, cutaneous inflammatory disorders, such as allergic contact dermatitis (183), atopic dermatitis (184–188), and psoriasis (183, 184, 189–191), are known to be promoted by the upregulation of selectins on dermal microvasculature. Specifically, P- and E-selectins are upregulated in skin lesions of cutaneous inflammatory patients, which enables inflammatory mononuclear phagocyte infiltration with subsequent release of soluble cytotoxic mediators, T cell activation, and destruction of the dermal layer of skin (154, 192–194). Atherosclerosis is another chronic inflammatory disease in which the recruitment of monocytes into selectin-expressing endothelial beds constitutes a key molecular event in the pathogenesis of the disease (195). Both E-and P-selectins are expressed on human arterial luminal endothelial cells of atherosclerotic plaques (195, 196), and mouse studies have shown that E-selectin and/or P-selectin deficiency substantially reduces the formation of atherosclerotic plaques, suggesting an overlapping function of these two selectins in the development of atherosclerotic lesions (197, 198). Murine classical monocytes (Ly6C^hi^) preferentially migrate into activated endothelium and infiltrate developing atheromas to become atherosclerotic macrophages, inflammatory DCs, or foam cells (139). Indeed, murine classical monocytes display high levels of PSGL-1 and higher binding affinity to E-/P-selectin expressing cells than do the non-classical patrolling monocytes, which might explain why they are preferentially recruited to sites of endothelial inflammation and thrombosis (138).

Inflammatory bowel disease (199–202), multiple sclerosis (203, 204), rheumatoid arthritis (205–207), and type 1 diabetes (208, 209) are other examples of inflammatory diseases in which upregulated E-/P-selectin expression in tissue microvasculature drives myeloid cell recruitment crucial for the development of the disease. Increased E-selectin expression and mononuclear phagocyte infiltration have been similarly observed in cases of transplantation rejection, including human renal (210, 211), lung (212–214), and cardiac (215–217) rejection and in acute graft versus host disease (218–221). Recruitment of inflammatory classical monocytes (222, 223) and pDCs (38, 41, 224) to tumor-cell-activated endothelium has also been reported to be dependent on E-selectin expression, which can lead to an inhibition of the tumor-specific immune defense response and induction of tolerance (225, 226). Some studies have also indicated that inflammatory monocytes license extravasation of tumor cells *via* the induction of E-selectin-dependent adhesive interactions (222).

## Therapeutic Strategies Targeting the Selectin/Selectin–Ligand Axis

The critical role of selectins and their ligands in the pathogenesis of many inflammatory conditions makes them potential molecular targets for therapy and, therefore, several strategies have been developed to interfere with this biology (182, 227, 228). One strategy relies on the development of pan-selectin competitive inhibitors that inhibit leukocyte/endothelial cell interaction and, therefore, cell recruitment to affected inflammatory or metastatic tissues. One example is the molecule Bimosiamose (Encysive Pharmaceuticals), a sLe^x^ mimetic that showed clinical efficacy in both asthma and psoriasis (229, 230). Other examples of pan-selectin inhibitors include sLe^x^-peptides (231), sLe^x^-bearing liposomes (232) or heparin oligosaccharides (233, 234). Also, GMI-127 and GMI-1271, E-selectin antagonists developed by GlycoMimetics based on the bioactive conformation of sLe^x^ in the carbohydrate-binding domain of E-selectin, have been used in treatment of sickle cell crisis (ClinicalTrials.gov Identifier: NCT00911495) and as an adjuvant for chemotherapy of hematological malignancies, including multiple myeloma and acute myeloid leukemia (NCT02811822, NCT02306291). In addition, an alternative approach involves the inhibition of the selectin ligand synthesis, either by interfering with the expression of key glycosyltransferases involved in sLe^x^ biosynthesis (235), or by using fluorinated analogs of Sia and/or Fuc residues (232, 236) that inhibit sLe^x^ synthesis. Finally, competing antibodies that bind to vascular E- and P-selectins and/or to selectin ligands expressed on the leukocyte cell surface constitute alternative methods that have been explored to prevent inflammatory exacerbation (182, 228). Indeed, a soluble form of PSGL-1 linked to the Fc portion of human IgG1 (PSGL-1-Ig) has been shown to inhibit leukocyte rolling in several disease models in mice (237–240).

On the other hand, the disruption of the selectin–leukocyte interaction has been reported to trigger severe immune-deficiency by disruption of immunosurveillance (241). Leukocyte adhesion deficiency Type II is a rare genetic disorder characterized by defective neutrophil and monocyte migration caused by a mutation in a GDP-Fuc transporter gene (242). This mutation leads to the formation of glycans that lack fucosylation, resulting in impaired leukocyte rolling and consequent leukocytosis and recurrent infections (242, 243). To overcome deficient cellular rolling, our lab developed a FT-driven sLe^x^ biosynthesis approach to enforce cell surface expression of E-selectin ligands while preserving cell viability, called glycosyltransferase-programmed stereosubstitution (GPS) (244, 245). This platform uses optimized reaction conditions, which enables the efficient α(1,3)-fucosylation of underfucosylatated sialylated type 2 lactosamine acceptors (Siaα2-3Galβ1-4GlcNAc), *via* a soluble α(1,3) FT, installing expression of sLe^x^ (Siaα2-3Galβ1-4GlcNAcα1-3Fuc) (3). GPS has been used to improve the recruitment of a variety of human and mouse cells into E-selectin-bearing endothelial beds, driving cell migration into bone marrow and inflammatory sites (123, 178, 208, 244, 246–248).

## Concluding Remarks

Specific migratory routes and distinct localization in steady and inflammatory conditions of the different human MPS subsets suggests that they play differential roles in immunity. These cells are operational in multiple beneficial processes, including immediate antimicrobial host defense, activation of the adaptive immune system, and tissue healing processes. However, they may also contribute to the pathobiology of several inflammatory conditions. A better understanding of the molecular basis of glycosylation-dependent creation of E-selectin ligands could yield the development of novel therapeutic approaches for inflammatory diseases, or, alternatively, could yield enhanced ability to infiltrate sites where immunity is needed (e.g., tumors or infection). To our knowledge, only a limited number of studies have analyzed the functional and structural biology of the full spectrum of E-selectin ligands expressed by different circulating human MPS subsets. Future work will be required to address this issue and to elucidate how custom-modified expression of these homing receptors can be achieved to preferentially influence the specific migratory routes of the different subsets of circulating MPS cells.

## Author Contributions

All authors listed have made a substantial, direct, and intellectual contribution to the work and approved it for publication.

## Conflict of Interest Statement

According to the National Institutes of Health policies and procedures, the Brigham and Women’s Hospital has assigned intellectual property rights regarding HCELL and GPS to the inventor (RS), who may benefit financially if the technology is licensed. RS’s ownership interests were reviewed and are managed by the Brigham and Women’s Hospital and Partners HealthCare in accordance with their conflict of interest policy. All other authors declare no competing financial interests.
